# Evaluation of Nephroprotective Activity of Berberis asiatica, Withania somnifera, and Their Combination in Streptozotocin-Nicotinamide Induced Diabetic Wistar Rats

**DOI:** 10.7759/cureus.79606

**Published:** 2025-02-25

**Authors:** Devkumar D Tiwari, Vandana M Thorat, Prathamesh V Pakale

**Affiliations:** 1 Department of Pharmacology, Krishna Institute of Medical Sciences, Krishna Vishwa Vidyapeeth (Deemed to be University), Karad, IND

**Keywords:** berberis asiatica, diabetic nephropathy, herbal therapy, nephroprotection, oxidative stress, renal function, withania somnifera

## Abstract

Background

Diabetic nephropathy (DN) is a major complication of type 2 diabetes mellitus, leading to end-stage renal disease. *Berberis asiatica* (BA) and *Withania somnifera* (WS) have demonstrated nephroprotective effects individually, but their combined potential remains unexplored. This study evaluates their individual and combined efficacy in improving renal function in streptozotocin-nicotinamide (STZ-NIC) induced diabetic rats.

Methods

A total of 78 adult Wistar rats were divided into 13 groups, including normal control (NC), diabetic control (DC), BA treated (250, 500, and 1,000 mg/kg), WS treated (250, 500, and 1,000 mg/kg), polyherbal combination (PHC) of BA and WS (250, 500, and 1,000 mg/kg), and standard drug-treated groups (metformin and glimepiride). Renal function markers (serum creatinine and urea) were measured, and histopathological analysis of kidney tissues was performed.

Results

Treatment with BA, WS, and PHC significantly reduced creatinine and urea levels compared to the DC group (p < 0.0001). The highest doses of BA (1,000 mg/kg) and WS (1,000 mg/kg) reduced creatinine by 14.5% and 15.8%, respectively, while PHC 1,000 mg/kg achieved a 21.1% reduction, comparable to standard drugs. Similarly, BA 1,000 mg/kg and WS 1,000 mg/kg reduced urea levels by 40.5% and 42.2%, respectively, whereas PHC 1,000 mg/kg exhibited the highest reduction of 55.9%, indicating a synergistic nephroprotective effect. Histopathological analysis confirmed reduced renal damage, with PHC 1,000 demonstrating nearly normal kidney architecture. The observed effects are attributed to the antioxidant, anti-inflammatory, and antihyperglycemic properties of BA and WS.

Conclusion

BA and WS at all doses showed significant nephroprotective effects. The combination of BA and WS (PHC 1,000) in equal amounts exhibited a synergistic effect, enhancing renal function and restoring kidney architecture in DN.

## Introduction

Diabetes mellitus (DM) is a chronic metabolic disorder characterized by persistent hyperglycemia due to insufficient insulin production or impaired insulin action. In particular, type 2 DM (T2DM) is a serious worldwide health issue, responsible for severe complications, including nephropathy (kidney damage), neuropathy (nerve damage), and cardiovascular diseases [1]. Among these complications, diabetic nephropathy (DN) remains a leading cause of end-stage renal disease (ESRD), affecting nearly 40% of diabetic patients worldwide [2].

Herbal medicine has gained substantial attention as an alternative or adjunct therapy for managing DM and its associated complications. Various medicinal plants consist of nephroprotective, antioxidant, and hypoglycemic characteristics, which make them excellent options for managing DM [3]. The nephroprotective and antidiabetic properties of *Withania somnifera* (WS) and *Berberis asiatica* (BA) have been extensively researched.

BA commonly known as Indian Barberry, is rich in bioactive alkaloids such as berberine, which has demonstrated antihyperglycemic, anti-inflammatory, and nephroprotective properties in diabetic models. It has been shown to reduce oxidative stress, regulate glucose metabolism, and improve renal function by decreasing creatinine and urea levels in diabetic rats [4,5].

Similarly, WS (ashwagandha) is a widely used adaptogenic herb and possesses strong antioxidant, anti-inflammatory, and hypoglycemic properties. Studies have demonstrated that WS significantly reduces hyperglycemia, restores renal markers, and mitigates oxidative stress-induced kidney damage in diabetic models [6,7].

Despite the individual therapeutic benefits of BA and WS, limited research has explored their combined effects in improving renal function in DM. Polyherbal combinations (PHCs) often exhibit synergistic effects, enhancing efficacy and reducing toxicity. Therefore, this study aims to investigate the nephroprotective potential of BA, WS, and their combination in streptozotocin-nicotinamide (STZ-NIC) induced diabetic rats.

By evaluating key renal biomarkers such as serum creatinine and urea, this study seeks to provide new insights into the potential synergistic benefits of these herbal extracts in managing DN. The findings could contribute to the development of safer and more effective herbal-based therapeutic strategies for DM and its associated renal complications. Notably, our previous study demonstrated the antidiabetic potential of BA and WS in STZ-NIC induced diabetic rats, confirming their role in glycemic control and renal protection [8].

## Materials and methods

Experimental animals

The study used adult albino Wistar rats of either sex (male/female), weighing 150-250 g and aged around 8-12 weeks. The animals were procured from the Central Animal House of Krishna Institute of Medical Sciences, Krishna Vishwa Vidyapeeth (KVV), Karad, Satara, India.

Facilities and housing conditions

The rats were held in a controlled laboratory environment with standardized air conditioning, maintaining a 12-hour natural light-dark cycle at an ambient temperature of 27-37°C. Throughout the research, they had unrestricted access to water and a standard pellet diet. The commercial pellet diet (VRK Solutions, Sangali, Maharashtra) consisted of 55% carbohydrates, 24% protein, 10% moisture, 5% fat, 4% fiber, 0.6% calcium, 0.3% phosphorus, and 9% ash (w/w). Approximately three to four rats were maintained in poly-acrylic cages (40 × 25 × 15 cm).

Ethical considerations

The Institutional Ethics Committee (IEC) of KVV (IEC Approval No. 385/2020-2021) provided ethical approval. The animal study was conducted following approval from the Institutional Animal Ethics Committee (IAEC) of KVV (Reg. No. 255/PO/REBi/S/2000/CPCSEA, IAEC Approval No. IAEC/KIMS/2021/16). All procedures were performed in compliance with the guidelines established by the Committee for Control and Supervision of Experiments on Animals (CCSEA).

Chemicals and drugs

All chemicals, drugs, and reagents used were of analytical grade.

PHC

A combination of BA and WS in a 1:1 ratio was used for the study. Standardized dried ethanolic root extracts of BA and WS were obtained from Natucare India Pvt. Ltd. and Bhagwati Herbal and Healthcare Pvt. Ltd., Vapi, India.

Induction of T2DM

**Figure 1:**
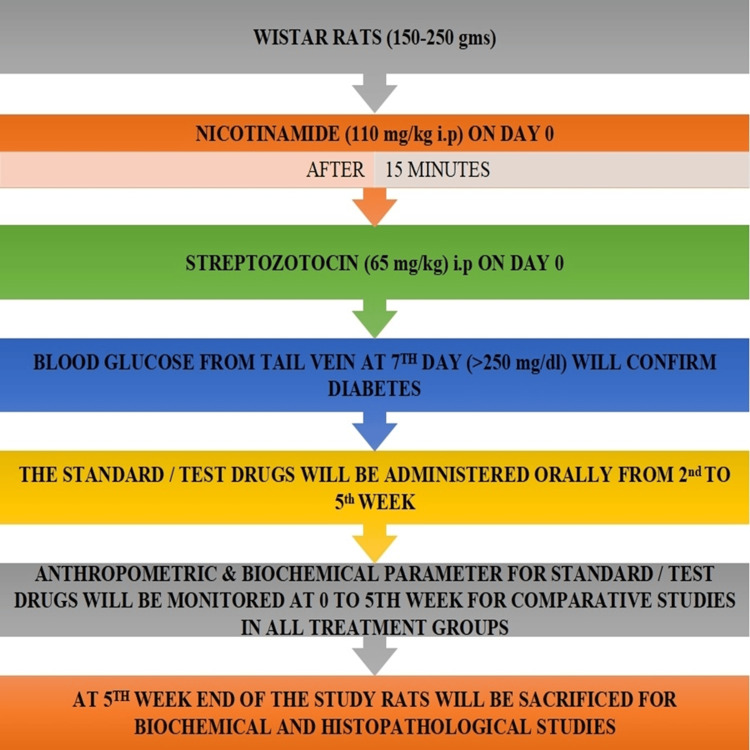
Induction of type 2 diabetes mellitus gms, grams; mg/kg, milligram/kilogram; i.p., intraperitoneally

STZ and NIC were procured in pure powder form from Sisco Research Laboratories (SRL) Pvt. Ltd., Mumbai, India. T2DM was induced by injecting a single dose of NIC (110 mg/kg BW) in physiological saline and 15 minutes later with a single dose of STZ (65 mg/kg BW) intraperitoneally (i.p.) dissolved in 0.01 M freshly prepared cold citrate buffer (pH 4.5) in overnight fasted rats. The rats were kept and monitored for seven days after the injecting STZ-NIC. Serum glucose estimations (blood sugar > 250 mg/dL) were undertaken periodically (day 0 to day 7) from the tail vein prick method using a glucometer to confirm the production of DM. Animals showing fasting blood glucose higher than 250 mg/dL were considered diabetic. Rats having random blood sugar levels of more than 250 mg/dL on the seventh day were considered diabetic and used for further studies (Figure 1).

Standard drugs

Metformin (MET) and glimepiride (GLI) were procured in pure powder form from Smurthi Organic Limited, Solapur, India.

Preparation of reagents

NIC was freshly prepared at 110 mg/kg body weight in 0.9% physiological saline. STZ was prepared at 65 mg/kg body weight in freshly prepared cold 0.1 M citrate buffer (pH 4.5). Citrate buffer (0.1 M) was prepared by dissolving 10.5 g citric acid and 14.7 g sodium citrate in 500 mL of distilled water. The final volume was adjusted to 1 L, and pH was set to 4.5 using hydrochloric acid or sodium hydroxide [8].

Experimental design

A total of 78 adult albino Wistar rats were randomly allocated into 13 experimental groups (six rats per group) and maintained for five weeks (Table 1).

**Table 1:** Experimental group design BA, Berberis asiatica; DC, diabetic control; GLI, glimepiride; MET, metformin; mg/kg, milligram/kilogram; mL/kg, milliliter/kilogram; NC, normal control; PHC, polyherbal combination; WS, Withania somnifera; STZ-NIC, streptozotocin-nicotinamide

Group No.	Group Name	Treatment	Dose (mg/kg, Oral)
1	NC	Distilled water	10 mL/kg
2	DC	STZ-NIC (diabetes induction) + distilled water	10 mL/kg
3	BA 250	Ethanolic root extract of BA	250
4	BA 500	Ethanolic root extract of BA	500
5	BA 1,000	Ethanolic root extract of BA	1,000
6	WS 250	Ethanolic root extract of WS	250
7	WS 500	Ethanolic root extract of WS	500
8	WS 1,000	Ethanolic root extract of WS	1,000
9	PHC 250	Ethanolic root extract of BA + WS (1:1)	250 (125+125)
10	PHC 500	Ethanolic root extract of BA + WS (1:1)	500 (250+250)
11	PHC 1,000	Ethanolic root extract of BA + WS (1:1)	1,000 (500+500)
12	MET	MET (standard drug)	250
13	GLI	GLI (standard drug)	10

Experimental model of T2DM

STZ-NIC Induced Diabetes Model

The diabetogenic effect of STZ was first reported by Rakieten et al. [9]. STZ exhibits selective cytotoxicity to pancreatic β-cells. Masiello developed the STZ-NIC model, which uses NIC to partially protect β-cells from STZ induced cytotoxic effects, resulting in a T2DM model [10,11].

Evaluation parameters

Biochemical Analysis

Blood samples were collected via cardiac puncture under light anesthesia at the end of the study for biochemical analysis.

Histopathological Examination

At the end of the experiment, rats were sacrificed, and kidney and liver tissues were immediately fixed in 10% buffered neutral formalin. The tissues were embedded in paraffin, sectioned at 5 µm thickness, and stained with hematoxylin and eosin (H&E). Microscopic analysis was performed to assess histopathological changes. Investigators conducting histological evaluations were blinded to the biochemical results and treatment allocations.

Statistical analysis

All the results were presented as mean ± standard deviation. Statistical analysis was performed using one-way analysis of variance (ANOVA) followed by a Tukey-Kramer multiple comparison test for post hoc analysis to find the difference between the means. A P-value of less than 0.05 was considered statistically significant; P represents the probability factor. All the statistical methods were carried out using the Statistical Package for the Social Sciences software version 29.0.0 (IBM Corp., Armonk, NY).

## Results

Effect of BA on renal function in diabetic Wistar rats

Ordinary ANOVA followed by the Tukey Kramer multiple comparison test (post hoc) revealed statistically significant differences in both the kidney functions (creatinine and urea) in all the study groups (NC, DC, BA 250, BA 500, BA 1,000, MET, and GLI) (P < 0.0001) (Table 2).

**Table 2:** Effect of BA on renal function in diabetic Wistar rats All the values are expressed in mean ± SD. P < 0.05 is considered significant. ^a^DC differs significantly from BA 250, BA 500, BA 1,000, MET, and GLI groups. ^b^MET differs significantly from DC, BA 250, BA 500, BA 1,000, and GLI groups. ^c^GLI differs significantly from DC, BA 250, BA 500, BA 1,000, and MET groups. ^1^P < 0.05. ^2^P < 0.01. ^3^P < 0.001. BA, Berberis asiatica; DC, diabetic control; F, f-statistic; GLI, glimepiride; MET, metformin; NC, normal control; P, probability value; SD, standard deviation

Groups (mg/dL)	NC	DC	BA 250	BA 500	BA 1,000	MET	GLI	F	P
Test									
Creatinine	0.59 ± 0.12^a3^	0.76 ± 0.04^b3c3^	0.68 ± 0.02	0.67 ± 0.01^a1^	0.65 ± 0.01^a2^	0.60 ± 0.01^a3^	0.6 ± 0.01^a3^	8.91	P < 0.0001
Urea	35.37 ± 0.5^a3^	90. 33 ± 1.75	69.42 ± 2.02^a3b3c3^	62.17 ± 1.17^a3b3c3^	53.75 ± 1.2^a3b3c3^	40.67 ± 1.2^a3^	40.17 ± 1.5^a3^	1165.2	P < 0.0001

**Figure 2:**
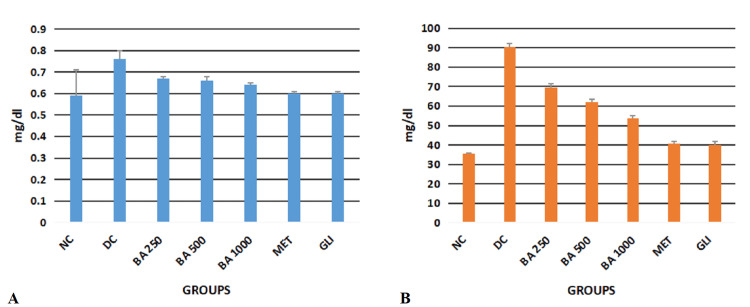
Graphical representation of effect of BA on renal functions in diabetic rats A, creatinine; B, urea; BA, Berberis asiatica, DC, diabetic control; GLI, glimepiride; MET, metformin; mg/dL, milligram/deciliter; NC, normal control

Post hoc analysis revealed statistically significant decreased levels of creatinine in the study groups BA 500 (P < 0.05), BA 1,000 (P < 0.01) NC, MET, and GLI (P < 0.001) when compared with the DC group, whereas statistically significant decreased levels of Urea were observed in all the study groups (NC, BA 250, BA 500, BA 1,000, MET, and GLI) when compared with the DC group (P < 0.0001). Post hoc test analysis revealed statistically significant increased levels of urea in the study groups BA 250, BA 500, and BA 1,000 when compared with the MET and GLI groups (P < 0.001) (Figure 2).

Effect of WS on renal function in diabetic Wistar rats

Ordinary ANOVA followed by the Tukey Kramer multiple comparison test (post hoc) revealed statistically significant differences in both the kidney functions tests (creatinine and urea) in all the study groups (NC, DC, WS 250, WS 500, WS 1,000, MET and GLI) (P < 0.0001) (Table 3).

**Table 3:** Effect of WS on renal function in diabetic Wistar rats All the values are expressed in mean ± SD. P < 0.05 is considered significant. ^a^DC differs significantly from WS 250, WS 500, WS 1,000, MET, and GLI groups. ^b^MET differs significantly from DC, WS 250, WS 500, WS 1,000, and GLI groups. ^c^GLI differs significantly from DC, WS 250, WS 500, WS 1,000, and MET groups. ^1^P < 0.05 ^2^P < 0.01 ^3^P < 0.001 DC, diabetic control; F, f-statistic; GLI, glimepiride; MET, metformin; NC, normal control; P, probability value; SD, standard deviation; WS, Withania somnifera

Groups (mg/dL)	NC	DC	WS 250	WS 500	WS 1,000	MET	GLI	F	P
Test									
Creatinine	0.59 ± 0.12^a3^	0.76 ± 0.04^b3c3^	0.67 ± 0.01	0.66 ± 0.02^a1^	0.64 ± 0.01^a2^	0.60 ± 0.01^a3^	0.6 ± 0.01^a3^	8.797	P< 0.0001
Urea	35.37 ± 0.5^a3^	90. 33 ± 1.75^b3c3^	68.45 ± 0.58^a3b3c3^	62.18 ± 1.1^a3b3c3^	52.17 ± 1.5^a3b3c3^	40.67 ± 1.2^a3^	40.17 ± 1.5^a3^	1509.4	P< 0.0001

**Figure 3:**
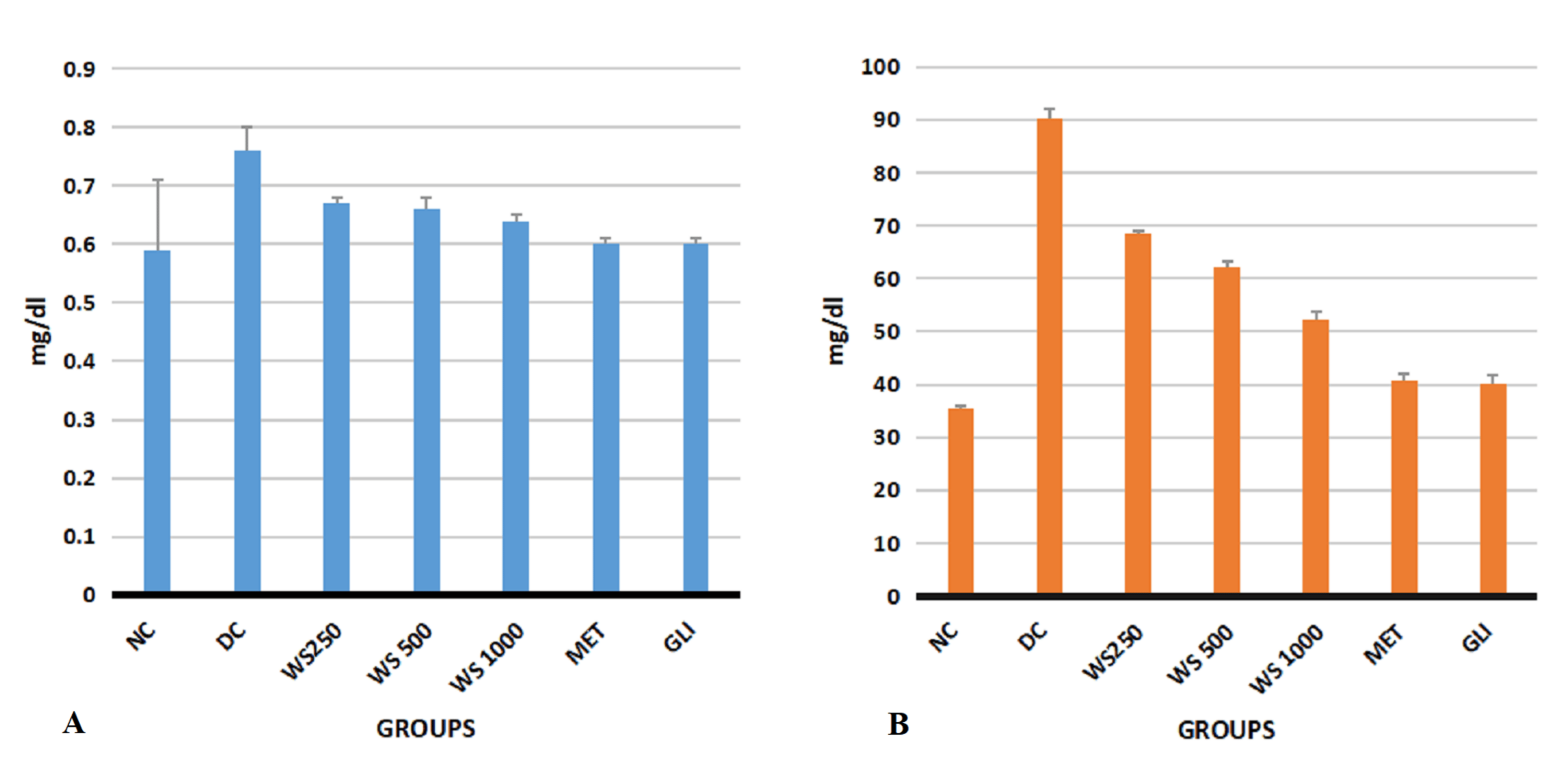
Graphical representation of effect of WS on renal functions in diabetic rats A, creatinine; B, urea; DC, diabetic control; GLI, glimepiride; MET, metformin; mg/dL, milligram/deciliter; NC, normal control; WS, Withania somnifera

Post hoc analysis revealed statistically significant decreased levels of creatinine in the study groups WS 500, WS 1,000, NC, MET, and GLI (P < 0.05) when compared with the DC group, whereas statistically significant decreased levels of urea were observed in all the study groups (NC, WS 250, WS 500, WS 1,000, MET, and GLI) when compared with the DC group (P< 0.001). Post hoc test analysis revealed statistically significant increased levels of urea in the study groups WS 250, WS 500, and WS 1,000 when compared with the MET and GLI groups (P < 0.001) (Figure 3).

Effect of PHC on renal function in diabetic Wistar rats

Ordinary ANOVA followed by the Tukey Kramer multiple comparison test (post hoc) revealed statistically significant differences in both the kidney functions tests (creatinine and urea) in all the study groups (NC, DC, PHC 250, PHC 500, PHC 1,000, MET, and GLI) (P< 0.0001) (Table 4).

**Table 4:** Effect of PHC on renal function in diabetic Wistar rats All the values are expressed in mean ± SD. P < 0.05 is considered significant. ^a^DC differs significantly from PHC 250, PHC 500, PHC 1,000, MET, and GLI groups. ^b^MET differs significantly from DC, PHC 250, PHC 500, PHC 1,000, and GLI groups. ^c^GLI differs significantly from DC, PHC 250, PHC 500, PHC 1,000, and MET groups. ^1^P < 0.05 ^2^P < 0.01 ^3^P < 0.001 DC, diabetic control; F, f-statistic; GLI, glimepiride; MET, metformin; NC, normal control; P, probability value; PHC, polyherbal combination; SD, standard deviation

Groups (mg/dL)	NC	DC	PHC 250	PHC 500	PHC 1,000	MET	GLI	F	P
Test									
Creatinine	0.59 ± 0.12^a3^	0.76 ± 0.04^b3c3^	0.66 ± 0.01^a1^	0.65 ± 0.01^a2^	0.6 ± 0.01^a3^	0.60 ± 0.01^a3^	0.6 ± 0.01^a3^	9.297	P< 0.0001
Urea	35.37 ± 0.5^a3^	90. 33 ± 1.75^b3c3^	64.5 ± 1.05^a3b3c3^	53 ± 2.3^a3b3c3^	39.83 ± 3^a3^	40.67 ± 1.2^a3^	40.17 ± 1.5^a3^	731.05	P< 0.0001

**Figure 4:**
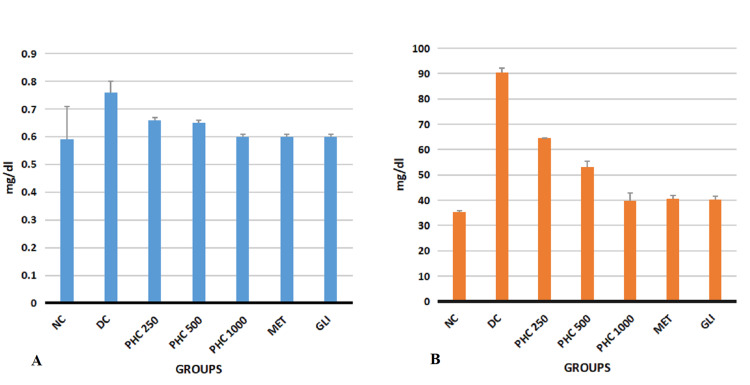
Graphical representation of effect of PHC on renal functions in diabetic rats A, creatinine; B, urea; DC, diabetic control; GLI, glimepiride; MET, metformin; mg/dL, milligram/deciliter; NC, normal control; PHC, polyherbal combination

Post hoc analysis revealed statistically significant decreased levels of creatinine in the study groups PHC 250 (P < 0.05), PHC 500 (P < 0.01), PHC 1,000, NC, MET, and GLI (P < 0.001) when compared with the DC group, whereas statistically significant decreased levels of Urea were observed in all the study groups (NC, PHC 250, PHC 500, PHC 1,000, MET, and GLI) when compared with the DC group (P < 0.001). Post hoc test analysis revealed statistically significant increased levels of urea in the study groups WS 250 and WS 500 when compared with the MET and GLI groups (P < 0.001) (Figure 4).

Histopathological effects of BA, WS, and PHC on renal tissue

Normal Control

**Figure 5:**
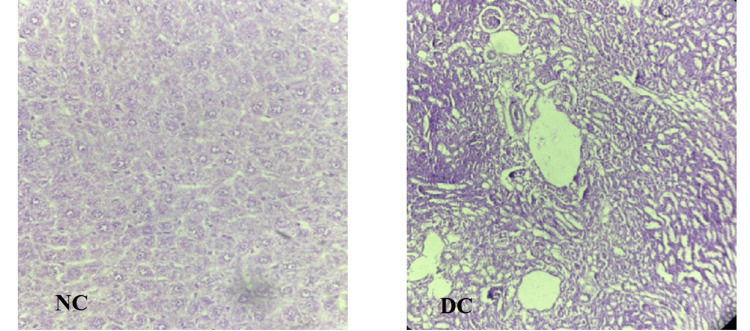
Histopathological examination of renal tissue of control groups NC, normal control; DC, diabetic control

Photomicrograph of kidney of the rats in the normal control (NC) group showed normal structure of the distal convoluted tubules that could be differentiated from the proximal convoluted tubules as having a larger and well defined lumina, and glomerulus. There was an absence of congestion of glomerular blood vessels, tubular necrosis, inflammation, cloudy degeneration, and hemorrhage in the NC group (Figure 5).

Diabetic Control

Photomicrograph of kidney of the rats in the diabetic control (DC) group rats showed cloudy changes in tubules, chronic inflammation, hypercellular glomeruli, and congestion of glomerular blood vessels (Figure 5).

Metformin

**Figure 6:**
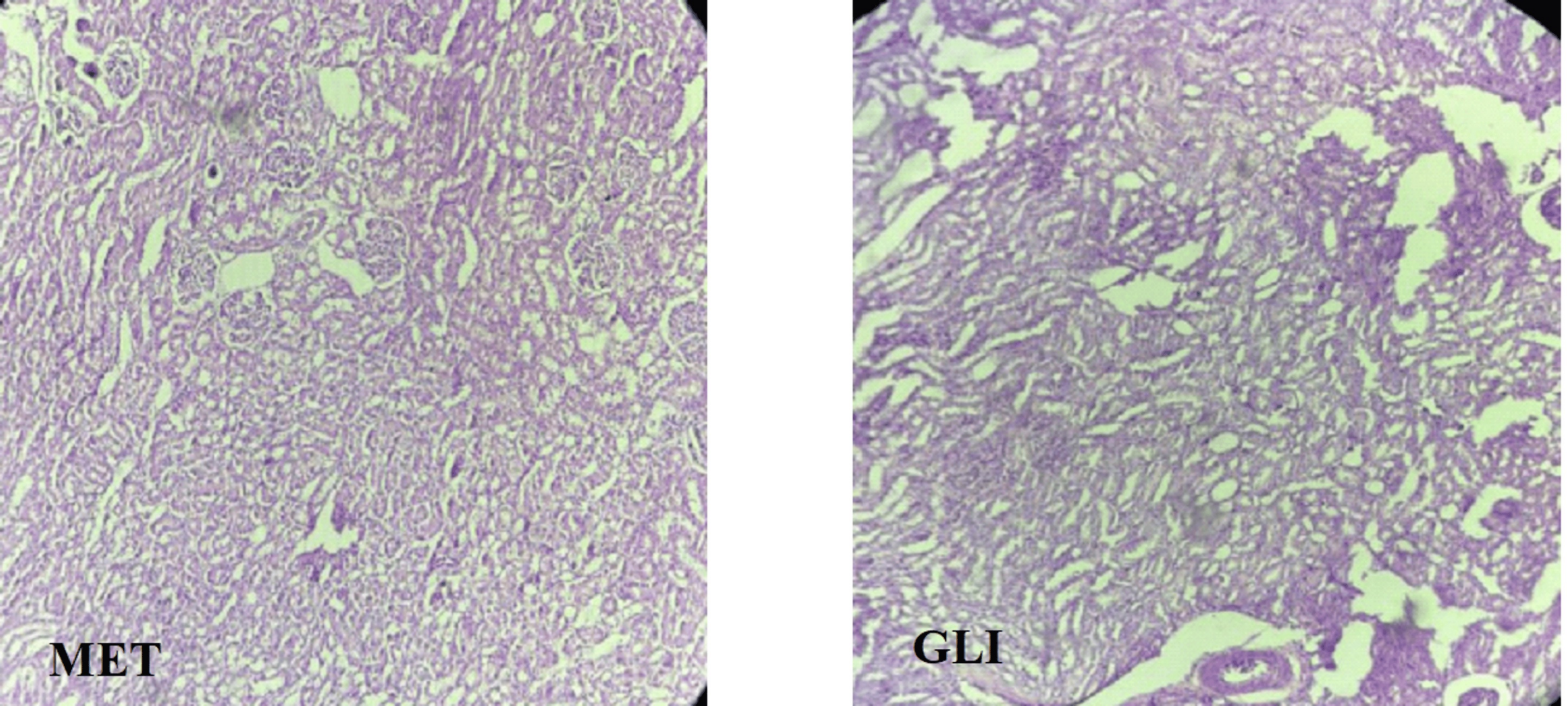
Histopathological examination of renal tissue of standard control groups MET, metformin; GLI, glimepiride

Photomicrograph of kidney of the rats in the MET group rats showed normal architecture of kidney, same as in the NC, BA, WS, and PHC 1,000 groups (Figure 6).

Glimepiride

Photomicrograph of kidney of the rats in the GLI group rats showed mild congestion of glomerular blood vessels (Figure 6).

BA 250

**Figure 7:**
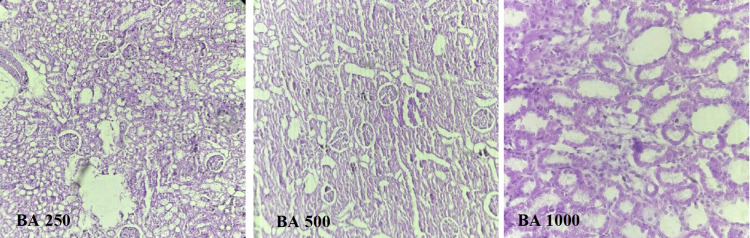
Histopathological examination of effect of BA on renal tissue of diabetic rats BA, Berberis asiatica

Photomicrograph of kidney of the rats in the BA 250 group showed cloudy changes in tubules, hypercellular glomeruli, and mild congestion of glomerular blood vessels (Figure 7).

BA 500

Photomicrograph of kidney of the rats in the BA 500 group rats showed focal cloudy changes in tubules (Figure 7).

BA 1,000

Photomicrograph of kidney of the rats of BA 1,000 group rats showed normal architecture of kidney same as in the NC group (Figure 7).

WS 250

**Figure 8:**
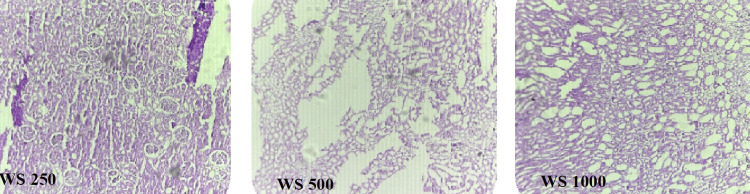
Histopathological examination of effect of WS on renal tissue of diabetic rats WS, Withania somnifera

Photomicrograph of kidney of the rats in the WS 250 group rats showed focal cloudy changes in tubules (Figure 8).

WS 500

Photomicrograph of kidney of the rats in the WS 500 group rats showed focal cloudy changes in tubules and hypercellular glomeruli (Figure 8).

WS 1,000

Photomicrograph of kidney of the rats in the WS 1,000 group rats showed normal architecture of kidney same as the NC and BA 1,000 groups (Figure 8).

PHC 250

**Figure 9:**
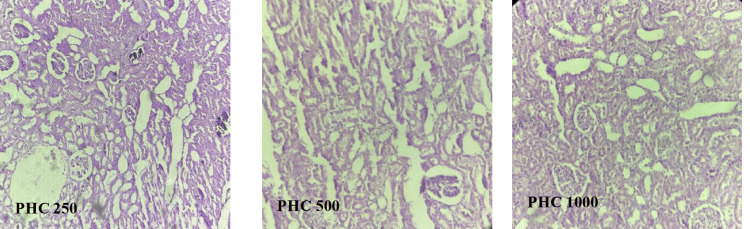
Histopathological examination of effect of PHC on renal tissue of diabetic rats PHC, polyherbal combination

Photomicrograph of kidney of the rats in the PHC 250 group rats showed mild congestion of glomerular blood vessels (Figure 9).

PHC 500

Photomicrograph of kidney of the rats in the PHC 500 group rats showed focal cloudy changes in tubules (Figure 9).

PHC 1,000

Photomicrograph of kidney of the rats in the PHC 1,000 group rats showed normal architecture of kidney same as in the NC, BA 1,000, and WS 1,000 groups (Figure 9).

## Discussion

T2DM is a global health burden associated with severe complications, including nephropathy [1]. DN remains a leading cause of ESRD, necessitating the exploration of alternative natural therapies to mitigate renal dysfunction and reduce harmful effects of current standardized drugs in a cost-effective manner [12]. Herbal interventions, such as BA and WS, have been traditionally used for their nephroprotective effects [13]. The current study investigates the potential of BA, WS, and their combination in improving renal function in STZ-NIC induced diabetic rats by evaluating key renal biomarkers such as serum creatinine and urea.

The hypoglycemic effects of BA and WS likely contribute to nephroprotection by improving glycemic control, which is crucial in preventing hyperglycemia-induced renal damage. Chronic hyperglycemia leads to oxidative stress, inflammation, and glomerular dysfunction, all of which accelerate the progression of DN. By lowering blood glucose levels, BA and WS help reduce these pathological stressors, thereby indirectly enhancing renal protection. The superior effects observed with PHC 1,000 suggest a synergistic mechanism where improved glycemic control further mitigates renal stress, reinforcing the role of BA and WS as potential adjunct therapies in managing DN.

The synergistic effects observed in the PHC 1,000 group are likely due to the interaction between bioactive compounds berberine (from BA) and withanolides (from WS). Berberine activates the AMPK pathway, enhancing glucose metabolism and reducing oxidative stress, while withanolides modulate the Nrf2 pathway, boosting antioxidant defense. This dual mechanism may contribute to the superior nephroprotective effect seen in the PHC 1,000 group, as evidenced by improved renal markers and histopathology. The nearly normal kidney architecture observed in this group suggests that the combination of BA and WS provides enhanced protection compared to individual treatments.

Creatinine

Creatinine is a crucial indicator of kidney function, and its elevated levels in diabetic conditions signify renal impairment [14]. In this study, DC group rats exhibited significantly higher creatinine levels compared to NC group rats, indicating impaired renal functions. Treatment with BA and WS resulted in a dose-dependent reduction in serum creatinine levels, with the highest doses (eg; BA 1,000 mg/kg and WS 1,000 mg/kg) demonstrating significant improvement. The PHC therapy, outstandingly PHC 1,000 group rats, showed further enhancement, suggesting a synergistic effect in restoring renal function.

Urea

Urea levels serve as another key biomarker for renal function, with increased levels in DM indicating reduced kidney efficiency in waste elimination [15]. The DC group rats displayed markedly elevated urea levels when compared to the NC group rats. The complete treatment with BA and WS significantly reduced urea levels in a dose-dependent manner, with the highest doses (BA 1,000 mg/kg and WS 1,000 mg/kg) exhibiting substantial improvement. The PHC 1,000 group demonstrated urea levels comparable to those of standard drug-treated groups (MET and GLI), reinforcing the potential nephroprotective synergistic effect of BA and WS.

The concept of synergy in polyherbal formulations has been widely recognized in traditional medicine and scientific research. Studies have demonstrated that the combined use of herbal extracts enhances efficacy while reducing toxicity, likely due to the complementary bioactive compounds targeting multiple pathological pathways. Previous research has shown that herbal combinations improve renal function by mitigating oxidative stress, regulating inflammatory markers, and enhancing glucose metabolism more effectively than individual plant extracts [16]. The present study aligns with these findings, as the combination of BA and WS (PHC 1,000) exhibited superior nephroprotective effects compared to their individual treatments.

Both BA and WS at higher doses (BA 1,000 mg/kg and WS 1,000 mg/kg) resulted in marked improvements in kidney function, reducing both creatinine and urea levels compared to the DC group when treated individually as well as in combination. The combination therapy (PHC 1,000) demonstrated even greater nephroprotective efficacy, suggesting a synergistic action through multiple molecular pathways. The study suggests that BA and WS effectively improve renal function by reducing creatinine and urea levels in diabetic rats. The superior efficacy of PHC 1,000 further supports the potential of herbal synergy, as previously observed in studies on polyherbal nephroprotective formulations [16]. Surprisingly, the PHC therapy, specifically PHC 1,000 group, exhibited superior efficacy, highlighting the possible synergistic effects of these herbal extracts. These outcomes support the therapeutic potential of BA and WS as alternative or adjunct treatments for DN.

Mechanistic insights on dose-dependent creatinine reduction

The observed dose-dependent reduction in creatinine levels may be attributed to the greater inhibition of oxidative stress and inflammation at higher doses of BA and WS. Increased oxidative stress is a key factor in DN, leading to renal damage and impaired creatinine clearance. Both BA and WS possess strong antioxidant properties, and at higher doses, they significantly upregulate endogenous antioxidant enzymes such as superoxide dismutase (SOD), catalase (CAT), and glutathione peroxidase (GPx), thereby neutralizing reactive oxygen species (ROS) and reducing lipid peroxidation [16]. Additionally, the anti-inflammatory effects of these herbs become more pronounced at higher doses, as they suppress key inflammatory mediators such as TNF-α, IL-6, and NF-κB, which play a role in renal fibrosis and glomerular injury [7]. This enhanced suppression of oxidative stress and inflammation may explain the superior nephroprotective effects observed in the BA 1,000, WS 1,000, and PHC 1,000 groups. Furthermore, the synergistic action of BA and WS in the PHC formulation likely potentiates these effects by targeting multiple pathways involved in renal protection [16].

The nephroprotective effects of BA and WS can be attributed to their antioxidant, anti-inflammatory, and anti-hyperglycemic properties. Berberine and withanolides, the major bioactive compounds in BA and WS, have been reported by multiple researchers to mitigate oxidative stress and inflammation, modulate glucose metabolism, and enhance endogenous antioxidant defense mechanisms, thereby exhibiting renoprotective effects [4-7]. The combination of these herbs likely exerts a synergistic effect by targeting multiple pathways involved in DN, thereby improving renal function more effectively than individual treatments [17].

The findings of our study on the effects of WS and BA align well with existing literature. For instance, WS has been shown to significantly reduce blood glucose levels in diabetic rats, as demonstrated by Utami et al. [18]. Similarly, the hypoglycemic effects of BA have been reported by Sok Yen et al. [19], which supports the blood sugar-lowering effects observed in our study. In terms of antioxidant activity [8], WS has been shown to enhance antioxidant enzyme levels in diabetic rats, as found by Khan et al. [16]. Likewise, the antioxidant effects of BA were corroborated by Sharma et al. [20]. With respect to kidney function, WS has been reported to improve renal markers such as creatinine and urea in diabetic models, aligning with our findings, as shown by Vedi et al. [21]. Additionally, BA has demonstrated nephroprotective properties in diabetic models, as confirmed by Ahmad et al. [22]. The combination therapy of WS and BA species has shown potential synergistic effects in improving glucose metabolism and renal function. A similar study on PHC was also evidenced by Reddy et al. [23]. The study conducted by Khan et al. [16] has also reported the nephroprotective synergistic effect of PHC, further reinforcing the benefits of the PHC treatment observed in our research. Similar synergistic effects of PHC therapy were also observed in the study conducted by Reddy et al. [23], which coincides with our study.

Limitations

While this study provides valuable insights, it is limited by its use of animal models, which may not fully replicate human liver pathology. Moreover, the study focuses on specific doses and markers, which may not capture the full spectrum of the herbs' effects. Future research should address these limitations and include a broader range of dosages, longer study durations, and clinical trials.

Future clinical studies will be necessary to evaluate the safety, optimal dosage, and efficacy of the BA and WS combination in diabetic patients. Such trials will also help assess potential pharmacokinetic interactions, long-term effects, and any unforeseen adverse reactions, ultimately determining the clinical relevance of this herbal intervention for managing DN.

## Conclusions

The study highlights the nephroprotective potential of BA and WS in DN. Both herbs individually exhibit the nephroprotective effect at all the different doses (250, 500, and 1,000). Their combination significantly reduced renal markers (creatinine and urea) in STZ-NIC induced diabetic rats, demonstrating a synergistic effect. The highest dose of the combination (PHC 1,000) showed superior renal protection, comparable to standard drugs (MET and GLI). Histopathological analysis further confirmed these protective effects, as PHC 1,000-treated kidneys exhibited normal renal architecture with minimal damage, similar to the NC group. The nephroprotective effects are attributed to the antioxidant, anti-inflammatory, and antihyperglycemic properties of BA and WS. These findings suggest that their combination could be a promising, natural, and economical alternative for managing DN.
